# Sleep-Wake States Are Encoded across Emotion Regulation Regions of the Mouse Brain

**DOI:** 10.1523/ENEURO.0291-25.2025

**Published:** 2025-10-31

**Authors:** Kathryn K. Walder-Christensen, Jack Goffinet, Alexandra L. Bey, Reah Syed, Jacob Benton, Stephen D. Mague, Elise Adamson, Sophia Vera, Hannah A. Soliman, Sujay Kansagra, David Carlson, Kafui Dzirasa

**Affiliations:** ^1^Department of Psychiatry and Behavioral Sciences, Duke University Medical Center, Durham, North Carolina 27710; ^2^Department of Computer Science, Duke University, Durham, North Carolina 27708; ^3^Department of Neurobiology, Duke University Medical Center, Durham, North Carolina 27710; ^4^Department of Biomedical Engineering, Duke University, Durham, North Carolina 27708; ^5^Departments of Neurology, Duke University Medical Center, Durham, North Carolina 27710; ^6^Departments of Biostatistics and Bioinformatics, Duke University Medical Center, Durham, North Carolina 27710; ^7^Departments of Electrical and Computer Engineering, Duke University, Durham, North Carolina 27708; ^8^Civil and Environmental Engineering, Duke University, Durham, North Carolina 27708; ^9^Howard Hughes Medical Institute, Chevy Chase, Maryland 20815; ^10^Department of Neurosurgery, Duke University Medical Center, Durham, North Carolina 27710

**Keywords:** amygdala, networks, sleep, stress

## Abstract

Emotional dysregulation is highly comorbid with sleep disturbances. Sleep is composed of unique physiological states that are reflected by conserved brain oscillations. Though the role of these state-dependent oscillations in cognitive function has been well established, less is known regarding the nature of state-dependent oscillations across brain regions that strongly contribute to emotional function. To characterize these dynamics, we recorded local field potentials simultaneously from multiple cortical and subcortical regions implicated in sleep and emotion regulation and characterized widespread patterns of spectral power and synchrony between brain regions during sleep-wake states in male and female mice. First, we showed that single brain regions encode sleep state, albeit to various degrees of accuracy. We then identified network-based classifiers of sleep based on the combination of features from all recorded brain regions. Spectral power and synchrony from brain networks allowed for automatic, accurate, and rapid discrimination of wake, non-REM sleep (NREM), and rapid eye movement (REM) sleep. When we examined the impact of commonly prescribed sleep-promoting medications on neural dynamics across these regions, we found disparate alterations to both cortical and subcortical activity across all three states. Finally, we found that a stress manipulation that disrupts circadian rhythm in male mice increased sleep fragmentation without altering the underlying average brain dynamics across sleep-wake states. Thus, we characterized state-dependent brain dynamics across regions canonically associated with emotions.

## Significance Statement

Sleep and emotion regulation are known to be intertwined at the level of behavior and in neuropsychiatric illnesses. Here, we examined how brain regions involved in emotion regulation encode wake and sleep states by performing multisite electrophysiological recordings in mice. We developed classifiers that rapidly labeled sleep-wake states from brain activity alone. We then identified how commonly prescribed sleep-inducing medications have unique impacts on brain activity throughout these emotion regulation regions. Finally, we explored the impact of circadian rhythm disruption on sleep architecture and brain activity. Together, these data shed light on how brain regions that regulate emotion behave during sleep so that one day treatments to improve both sleep and emotional well-being may be developed.

## Introduction

Sleep is composed of fundamental states with distinctive behavioral, physiological, and neurophysiological signatures that are conserved across mammalian species (Lo et al., 2004; Keene and Duboue, 2018). Wake is a state of behavioral arousal with low-amplitude, mixed-frequency brain activity and high muscle tone. Sleep is a reversible state of behavioral quiescence, which can be further decomposed into rapid eye movement (REM) sleep, defined by low-amplitude, mixed-frequency oscillations with increased theta and gamma brain activity and absence of muscle tone, and non-REM (NREM) sleep, defined by high-amplitude delta-frequency brain activity and low muscle tone (Aserinsky and Kleitman, 1953; Kohn et al., 1974; Brown et al., 2012). Inadequate timing, quality, or quantity of sleep affects many aspects of health and disease, from cognition (Killgore, 2010) to immune function (Besedovsky et al., 2019). Impairment of sleep is a common and debilitating symptom shared across numerous neuropsychiatric disorders, especially in disorders of emotion regulation (Baglioni et al., 2016). Overlap in the regulation of sleep and mental well-being has been identified from the level of genes (e.g., genome-wide association studies linking molecular clock genes to bipolar and major depressive disorders; Jagannath et al., 2017) to population-level epidemiology (e.g., higher rates of depression in night-shift workers; Torquati et al., 2019). In experimental models, manipulating sleep quantity or quality can alter emotion regulation (Goldstein and Walker, 2014). There is evidence to suggest that sleep and emotion regulation overlap also occurs at the level of brain networks (Mu and Huang, 2019), though limited studies examine how emotion regulation brain networks behave during sleep states or how sleep-altering treatments impact these brain dynamics.

There has been significant progress, largely in rodent models, in understanding how different populations of neurons distributed throughout the brain exert widespread effects to regulate transitions into, or maintenance of, sleep-wake states, including forebrain cholinergic neurons, hypothalamic histaminergic and orexinergic neurons, and brainstem serotonergic, dopaminergic, and noradrenergic neurons (Lu et al., 2006; Brown et al., 2012; Mahoney et al., 2019). These distributed ensembles of neurons work together to organize sleep architecture. Indeed, several studies using parallel recordings from multiple brain regions have demonstrated the utility of studying the regulation of sleep across distributed brain regions (Gervasoni et al., 2004; Takahashi et al., 2009; Zhang et al., 2022). Recent work using single- and multiregion electrophysiological data in mice demonstrated the ability to decode sleep-wake states using only milliseconds of neural activity (Parks et al., 2024). Though this study was largely limited to decoding patterns within (but not across) subjects, these findings indicated the robust signatures of sleep across the brain (Parks et al., 2024).

Similarly, the brain regions implicated in emotion and mood regulation are numerous and widely distributed, including cortical, limbic, striatal, midbrain, and brainstem areas (Nestler et al., 2002; Hultman et al., 2016, 2018). There is notable overlap in various neuromodulatory systems regulating emotion and sleep, including cholinergic neurons, dopaminergic neurons, serotonergic neurons, and noradrenergic neurons, which may indicate shared neuronal networks underlying regulation of emotions and sleep-wake biology (Dzirasa et al., 2006; Brown et al., 2012; Mu and Huang, 2019). Here, we focused on a set of eight regions across the brain that are conserved between rodents and humans, implicated in emotional regulation, and are reliably accessible using standard electrophysiological microwires (Mague et al., 2022; Hughes et al., 2024). Specifically, we recorded from the cingulate cortex (CxCg), prelimbic cortex (CxPrL), infralimbic cortex (CxIL), nucleus accumbens (core and shell grouped together; NAc), amygdala (basolateral and central grouped together; Amy), medial dorsal thalamus (ThalMD), ventral hippocampus (HippV), and ventral tegmental area (VTA). All these regions, either in isolation or as components of neural circuits, have also been implicated in sleep biology. For example, basolateral Amy is critical for the transition between NREM and REM (Hasegawa et al., 2022) and lesions of the central nucleus of the Amy decreased REM (Tang et al., 2005). CxCg is associated with awakening in insomnia (Guo et al., 2023; Li et al., 2024), lesions in the prefrontal cortex including PrL and IL are associated with decreased REM sleep and increased sleep fragmentation (Chang et al., 2014), and pyramidal neurons in IL have their highest activity during REM (Hong et al., 2024). The core region of NAc can be activated to induce NREM sleep (Oishi et al., 2017), and lesioning of NAc core and shell increases wakefulness (Qiu et al., 2012). Lesion of ThalMD increased wakefulness in cats (Marini et al., 1988) but decreased wakefulness in rats (Sriji et al., 2021). HippV plays a critical role in emotional memory consolidation during sleep (Pronier et al., 2023). Finally, GABAergic neurons in VTA promote NREM sleep (Chowdhury et al., 2019). Though these findings clearly establish the role of these individual brain regions in sleep, it remains unclear how these regions are modulated in concert across sleep-wake states. Addressing such a question requires studies examining neural activity at the seconds-to-minutes timescale, which comprises the typical duration of a sleep state for mice and humans (Arrigoni and Saper, 2014). Such studies have been lacking, as prior work has largely focused on sleep-related molecular and cellular activity on the millisecond-to-second timescale or circadian rhythms of sleep-wake behavior on the hours-to-days timescale.

Here we begin to close this gap by characterizing the brain oscillatory patterns that are reliably observed within and across emotional regions during sleep-wake states. We achieved this by applying supervised machine learning techniques to local field potentials (LFPs) recorded simultaneously from multiple brain regions in mice (Hultman et al., 2018; Mague et al., 2022; Walder-Christensen et al., 2024). This approach yields electrical functional connectome-based (electome) networks, which integrate widespread neural activity from the milliseconds-to-minutes timescale. The activity of an electome network, defined by the coexpression of its component regions across seconds of time, can then be used for brain state classification. Thus, this approach enables brain states to be rapidly determined from long-term recordings (Talbot et al., 2023). We trained our electome-based classifier based on power and cross-power spectral density estimation (a measure of synchrony between two regions for a specific frequency) in eight cortical and subcortical brain regions, and we validated it against electromyography (EMG) and brain-based measures traditionally used for distinguishing sleep-wake states. Finally, we characterized the impact of pharmacological manipulations, commonly prescribed for emotional dysregulation and sleep, on regional brain dynamics, and we investigated the impact of chronic stress on sleep brain dynamics using the EMG-free sleep-wake classifier.

## Materials and Methods

### Mice and husbandry

All care and experimental procedures were conducted according to IACUC-approved protocols at Duke University. Male and female C57BL/6J (C57; JAX strain 000664) mice purchased from the Jackson Laboratory were used to train (*n* = 9 male) and validate [*n* = 5 male (mice utilized again for circadian stress)/4 female] the model and for psychopharmacology (*n* = 5 male mice) and circadian stress manipulation experiments (*n* = 9 male mice). Mice were maintained in single-sex cages with 12 h light/dark cycle (except for animals undergoing chronic light stress, see below) with food and water *ad libitum*. Mice were group-housed (≤5 mice per cage) except during habituation and sleep recordings (see below). Studies were conducted with mice aged 9–30 weeks, and mice were recorded in a pseudo-home cage (see below for details). Animals were excluded if electrode tracts were identified outside of the targeted regions; four mice were excluded based on this criterion from pharmacological manipulation study and are not included in the *n* reported for the analysis. Mice requiring EMG were excluded if EMG power signal was visibly saturated across all frequencies. Five mice were excluded based on this criterion: one from the training dataset and four from the chronic stress implanted dataset, both of which are not included in the *n* reported for the analysis. Three mice did not survive the surgery or postoperative period and were thus not included in the *n* reported for the analysis.

### Electrode implantation surgery

Thirty-nine mice (ages 8–12 weeks) were anesthetized with 1% isoflurane, placed in a stereotaxic device, and metal ground screws were secured above the cerebellum and anterior cranium. The recording bundles designed to target basolateral and central amygdala (Amy), medial dorsal thalamus (ThalMD), nucleus accumbens core and shell (NAc), ventral tegmental area (VTA), medial prefrontal cortex (mPFC), and ventral hippocampus (HippV) were centered based on stereotaxic coordinates measured from bregma (Amy: −1.4 mm AP, 2.9 mm ML, −3.85 mm DV from the dura; ThalMD: −1.58 mm AP, 0.3 mm ML, −2.88 mm DV from the dura; VTA: −3.5 mm AP, ±0.25 mm ML, −4.25 mm DV from the dura; HippV: −3.3 mm AP, 3.0 mm ML, −3.75 mm DV from the dura; mPFC: 1.62 mm AP, ±0.25 mm ML, 2.25 mm DV from the dura; NAc: 1.3 mm AP, 2.25 mm ML, −4.1 mm DV from the dura, implanted at an angle of 22.1°). To target layers 2/3 of cingulate, prelimbic, and infralimbic cortex, a mPFC bundle was assembled by building a 0.5 and 1.1 mm DV stagger into the electrode bundle microwires. NAc core and shell were targeted in a single bundle with a 0.6 mm DV stagger. Basolateral and central amygdala were targeted by building in a 0.5 mm ML + 0.25 mm DV stagger into the Amy bundle. Animals were implanted bilaterally in mPFC and VTA. All other bundles were implanted in the left hemisphere. A 20 μm wire was inserted into the trapezius muscle to record muscle activity for a subset of mice used to acquire ground truth sleep labels along with multisite electrophysiology. A subset of the mice had additional brain regions recorded that were not used in these studies. Five of the male mice used to generate the training dataset had wires placed in dorsal hippocampus. All of the chronic circadian stress mice (which were also used as out-of-sample validation mice) had EMG and wires targeting anterior insular cortex, dorsal hippocampus, orbital frontal cortex, dorsal lateral striatum, dorsal medial striatum, lateral habenula, motor cortex, sensory cortex, substantia nigra pars reticulata, and visual cortex. To mitigate pain and inflammation related to the procedure, all animals received carprofen (5 mg/kg, s.c.) injections once prior to surgery and then daily for 3 d following surgery.

### Electrode placement confirmation

Histological analysis of implantation sites was performed at the conclusion of experiments to confirm recording sites used for neurophysiological analysis. Animals were perfused with 4% paraformaldehyde (PFA), and brains were harvested and stored for 24 h in PFA. Brains were sliced at 40 μm using a vibratome (Leica) and stained using DAPI (ab104139, Abcam) in the mounting media. Images were obtained using an Olympus Slide Scanner (VS200) using a 4× objective.

### Neurophysiological data acquisition

Similar to our prior work (Mague et al., 2022), mice were connected to either an M or mu headstage (Blackrock Microsystems) and placed into a cage with food and water. Mice were habituated to single housing for 2 d prior to recording. Mice were given extra enrichment including a chew bone, a wooden block, and a cup to hold food pellets.

On the day of recording, mice were placed into new cages with a small amount of bedding from their previous cage and allowed to acclimate for 3–6 h. Subsequently, neural activity was sampled at 30 kHz using the Cerebus acquisition system (Blackrock Microsystems) for at least 24 h inclusive of a full light/dark cycle. LFPs were bandpass filtered at 0.5–250 Hz and stored at 1,000 Hz. An online noise cancellation algorithm was applied to reduce 60 Hz artifact. Only the final 24 h of recording were utilized in the subsequent analyses. Neurophysiological recordings were referenced to a ground wire connected to ground screws in the skull.

### Sleep/mood medication exposure

After surgical recovery of at least 2 weeks, implanted mice were singly housed for 2 d prior to recording. Mice were recorded for 24 h following acute administration of a medication via intraperitoneal injection (diazepam 3 mg/kg or trazodone 40 mg/kg solubilized in 10% DMSO + 0.9% saline). Mice were given a 1 week washout period before administration of the other medication or vehicle control (saline containing 10% DMSO). Power and synchrony features (see below, LFP preprocessing and feature generation, for calculation of cross-power spectral density estimates used as the measure of synchrony) during the initial 2 h were averaged for each sleep-wake state based on network-classification labels.

### Chronic stress induction by aberrant light: dark cycle

Mice were singly housed and connected to the electrophysiology recording system. The 24 h prior to any change in their light cycle is considered the baseline recording [12 h light:12 h dark (T24)]. Then, the mice were exposed to chronic aberrant light interruption using a 3.5 h:3.5 h light dark (T7) cycle for 14 d (LeGates et al., 2012). After 14 d, the mice were immediately returned to the T24 light condition, and the following 24 h was analyzed for the poststress session. See Figure 6*A,B* for the schematic representation of the experimental paradigm.

### LFP preprocessing and feature generation

Power spectral density plots were calculated for each contact and inspected manually for the removal of poor channels (i.e., saturated). Outlying samples from each remaining channel were identified automatically as those points in a 30 Hz high-passed signal with an absolute value above 20 median absolute deviations of the channel’s activity. Individual channels in the same region of interest were then averaged in the time domain (NAc core and shell were averaged together and basolateral and central amygdala were averaged together). Then the LFPs were segmented into 2 s windows and cross-power spectral densities were calculated using Welch’s method independently for each window, both within regions (power) and between regions (cross-power spectral density estimate). Cross-power spectral density estimates quantify the extent to which two signals share energy at a given frequency. In the remainder of the paper, we use the term synchrony to refer to cross-power spectral density estimations. Twenty-nine frequency bins linearly spaced between 0 and 54.7 Hz were collected. Power and synchrony features were normalized by their median value over all frequencies and regions, within each mouse for each recording.

### EMG-informed cluster-based classification

**Figure 1.**
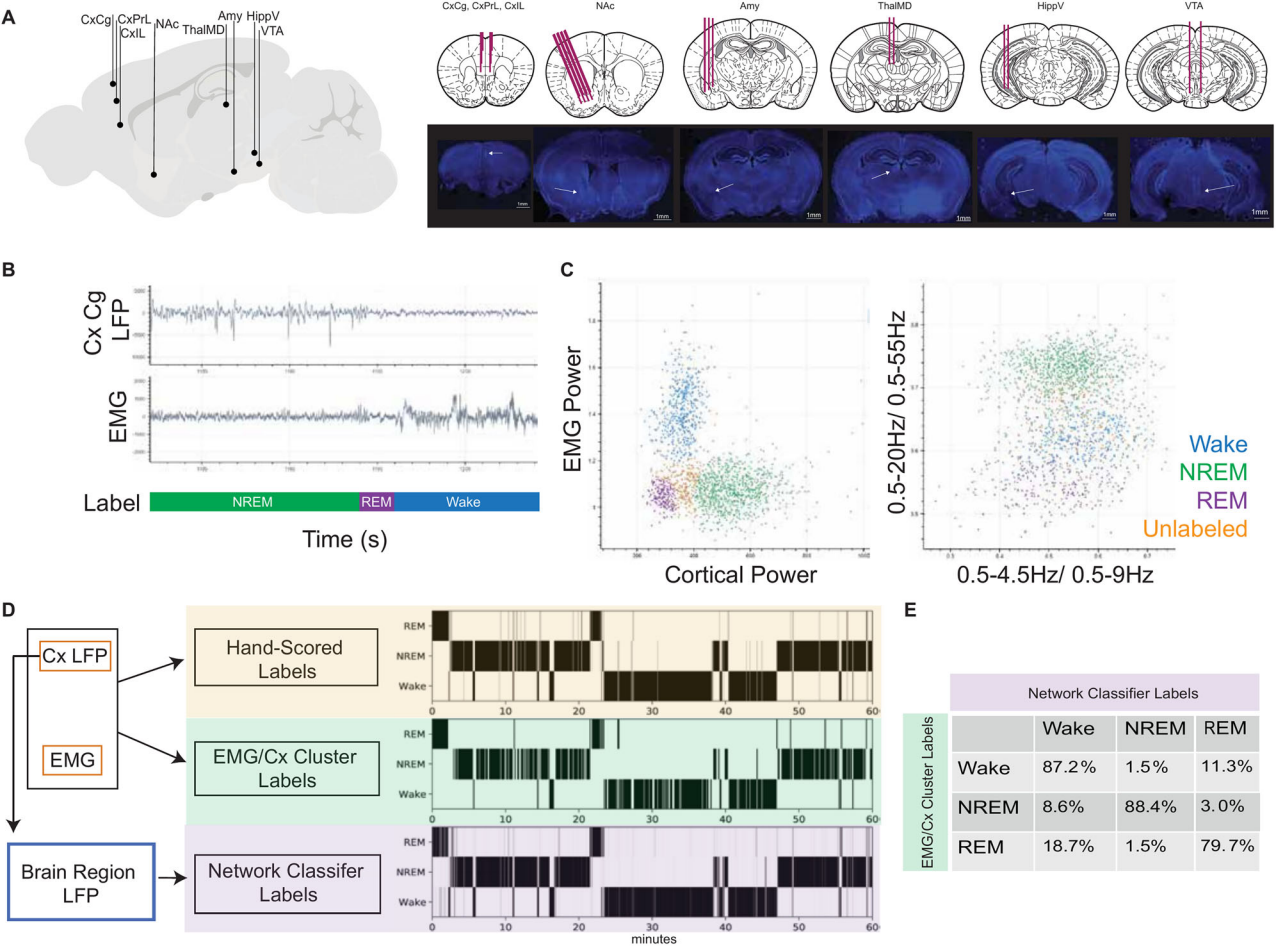
LFP-only network-based sleep labels recapitulate EMG-based labels. ***A***, Recording sites and representative histological targeting images from C57BL6/J mice used for network training and recording. ***B***, Representative LFP (from cingulate cortex, CxCg) and EMG traces and corresponding labels identified by hand. Green indicates NREM, purple indicates REM, and blue indicates wake. ***C***, Representative behavior state maps used for EMG-informed cluster-based sleep-wake state labels. Trapezius EMG power (*y*-axis) and root mean square of Cx_Cg (left) and cortical power ratios (*x*-axis, 0.5–4.5 Hz/0.5–9 Hz; *y*-axis, 0.5–20 Hz/0.5–55 Hz; right) used to manually identify wake (blue), NREM (green), and REM (purple) states. Unlabeled points are gold. ***D***, Diagram of input data used for three labeling methods and hypnodensity plots for a 1 h segment showing classification of wake, NREM, and REM based on hand-scored labels (top), EMG + Prelimbic cortex cluster-based labels (middle), and network-based classifier labels (bottom). Intensity of shading indicates label confidence (e.g., agreement between human raters or network-model label probabilities). ***E***, Confusion matrix of EMG + Prelimbic Cortex (EMG/Cx) cluster-based labels in comparison with network-based classifier labels. Correct network labeling in comparison with EMG/Cx Cluster label percentages are seen in the diagonal for wake, NREM, and REM, respectively. Misclassification is reported in the off-diagonal. Misclassification is highest between wake and REM.

A graphical interface was developed to annotate 2 s windows into wake, REM, and NREM sleep bouts by two-dimensional state mapping of theta, gamma, and delta power from single-channel LFPs with EMG atony used to delineate overlapping REM versus wake recordings based on previously defined electrophysiological properties (Gervasoni et al., 2004; Fig. 1*B*,*C*). EMG power was calculated as the sum of the signal’s average power per frequency bin in the 30–60 Hz band and in the 60–250 Hz band and was plotted on the *y*-axis (Dzirasa et al., 2006; Fig. 1*C*, left). Root mean square of LFP power was based on a single selected electrode site—here we use CxPrL, ventral hippocampus, or dorsal hippocampus—and was plotted on the *x*-axis (Fig. 1*C*, left). The wake cluster (blue) was identified based on high EMG power, the NREM cluster (green) was based on low EMG but high LFP power, and the REM cluster (purple) was based on low EMG power and low LFP power (Fig. 1*C*). Manual discrimination of clusters was done by drawing a polygon around the cluster and assigning it to a particular state, after which a user manually circles the three regions corresponding to wake, NREM, REM, and unknown sleep-wake states. A second plot based on spectral LFP power ratios [*x*-axis: (0.5–4.5 Hz)/(0.5–9 Hz); *y*-axis: (0.5–20 Hz)/(0.5–55 Hz)] was plotted (Fig. 1*C*, right). Clustering was refined using the second plot where the upper cluster (high power in <4.5 Hz frequencies) was defined as NREM. We observed high mixing of wake and REM in the bottom cluster and thus it was not used to distinguish between those states. All points that were outside of the hand-drawn polygons were considered unlabeled (gold) and were not used to calculate the accuracy of the EMG-free classifiers. Clusters were identified for data in 60 min segments to allow for optimal balance of labeling efficiency and discriminability between clusters.

### Hand-labeling of sleep labels

The EMG-informed cluster labels were validated against expert hand-labeling of sleep-wake states for a subset of recordings utilizing LFP traces from prelimbic or cingulate cortex, HippV, and EMG (Fig. 1*B*). Individual 30 or 60 min recordings were inspected and annotated with sleep-wake state labels based on heuristics established in the literature (Silber et al., 2007): low-amplitude, mixed-frequency brain oscillations along with increased muscle power in wake; high-amplitude, delta-predominant activity in cortex and HippV with low muscle power in NREM sleep; and low-amplitude, mixed-frequency oscillations with increased theta and gamma power in cortex and HippV with very low muscle power in REM sleep epochs, which were preceded by NREM epochs. Hand labels were annotated with a granularity of 2 s to match the automated sleep classifier and were chosen to optimally minimize multilabeled windows. Example labels of each state, transitions, and difficult-to-classify states were discussed with a sleep medicine specialist prior to establishing scoring rules. There was no coordination between the labelers after establishing the scoring rules.

### Interrater agreement

Three raters classified sleep-wake states for each 2 s window in four 1 h recordings of mouse LFPs and EMG. For this evaluation, there was no option to leave a window unclassified. The interrater agreement was quantified by calculating Cohen’s kappa for each pair of label sets using scikit-learn's cohen_kappa_score function.

### Supervised autoencoder sleep-wake state classifier

A supervised autoencoder (SAE) model was trained to predict EMG-informed cluster-based sleep labels (wake vs NREM vs REM) in 2 s windows. This follows the logic and methods described by Talbot et al. (2023). The SAE converts the observed neural data to a low-dimensional feature representation that both accurately summarizes the neural data and is predictive of sleep-wake state. The parameters are learned to both predict well and reconstruct the observed neural features. More specifically, the SAE minimizes the following objective:

L(C(Ax+b),y)+μ‖Hsoftplus(Ax+b)−x‖,

where x is the vectorized neural features (power and synchrony) associated with a single window and the matrix A and vector b define a linear transformation from the high-dimensional neural feature space (*d* = 1,856) to a low-dimensional summary feature representation (*d* = 32), termed “electome scores.” The first term in the objective is the prediction loss, where L(a,y) denotes cross-entropy loss with logits a and sleep-wake state label vector y and C is a diagonal matrix with three rows, one for each sleep-wake state. The second term in the objective is the reconstruction loss, for which the summary features (electome scores) are rectified with a softplus nonlinearity and subsequently projected to the high-dimensional neural feature space by the non-negative matrix H, defining the generative model associated with non-negative matrix factorization. *μ* > 0 determines the importance of the reconstruction relative to sleep label prediction. Each row of H is a non-negative vectorized rank-1 tensor (regions-by-regions-by-frequencies) that associates each electome score with a factor, termed an “electome factor,” in the high-dimensional neural feature space. The first three rows of H are termed the supervised electome factors because they correspond to each of the three sleep-wake state logits and can therefore be used to interpret the neural features that discriminate between the sleep-wake states, whereas the remaining rows are unsupervised electome factors. It is common for SAEs to use complex mappings defined by multilayer neural networks to map neural features to electome scores (Talbot et al., 2023), but here we use a simple linear mapping with a nonlinearity because we found this to be sufficient to obtain good performance.

To describe the classification performance of the SAE, we report balanced accuracy, which is the average recall of the classifier across all three sleep-wake states. This metric weights the classification of each of the three sleep-wake states equally, not in proportion to the portion of time spent in each state.

We used leave-one-mouse-out nested cross-validation to perform model selection, which uses two levels of cross-validation. While we later evaluate the final chosen model on new hold-out animals, we choose to use a nested cross-validation ensure an unbiased generalization estimate (Krstajic et al., 2014) from the initial set of nine training mice as well. Specifically, we rotated over the test mouse and then used three randomly shuffled splits of six training mice and two validation mice from the remaining eight mice and considered hyperparameter settings of *μ *= 10^−1^, 10^−2^, 10^−3^ for the interior loop of the nested cross-validation. After obtaining an unbiased estimate of performance and selecting the hyperparameters, we retrained on all nine mice with the chosen setting. This is the only model considered subsequently (and had no access to the rest of the validation studies during training). The same nested cross-validation procedure was used when considering the single-region models.

### Sleep architecture analyses

To integrate temporal information into the SAE’s per-window label prediction, a top-*k* Viterbi algorithm was applied to the label sequences. This method takes as input a transition matrix that describes the probability of transitioning from a given state to every other state in the next window (Viterbi, 1967). This matrix was estimated using several hours of hand-labeled sleep-wake states and collecting the empirical state transitions from each 2 s window to the next. The top-*k* Viterbi algorithm then gives the *k* most likely state sequences, given the SAE-produced per-window state probabilities and expected transition statistics. Sleep bout and transition statistics were calculated using these “smoothed” label sequences with *k* = 10.

### Summarizing LFP features that distinguish experimental conditions

To compare LFPs within the same sleep-wake state, but across various experimental conditions (male vs female, drug vs vehicle, and before vs after chronic stress), we test for statistical differences of LFP features across the conditions while controlling the familywise error rate at approximately *α* = 0.05 using corrected harmonic mean *p* values (HMPs; Wilson, 2019). This method, although not currently a standard practice, can test for differences in two samples in a way that is relatively insensitive to combining large numbers of tests (1,856 in this case), allowing us to test for differences between groups without first combining power features into a small number of hand-picked frequency bands. The HMP approach is also robust to dependencies between tests, for example, those comparing power features in nearby frequency bins, and maintains reasonable statistical power. More specifically, we perform independent two-sided *t* tests to compare the within-mouse averages of each LFP feature (power and synchrony) across windows. We then combine the *p* values and calculate a “headline” *p* value associated with the null hypothesis that the LFP features are the same across condition using the corrected HMP, following the procedure described by Wilson (2019). In comparison to procedures such as Benjamini–Hochberg that control the false discovery rate, the HMP is more sensitive in detecting significant groups of hypotheses at the expense of being less sensitive in detecting significant individual hypotheses (Wilson, 2019). For this reason, we visualize the individual features with statistically significant differences between conditions by plotting only the mean differences in features that reach unadjusted significance at *α* = 0.01, without regard to any HMP.

### Experimental design and statistical analysis

Data was plotted and analyzed in GraphPad Prism 9.1 and custom Python scripts as described above. Sleep architecture comparisons for sleep medication were analyzed using RM-ANOVA followed by Holm–Šídák's multiple-comparison test, and chronic light stress data was analyzed using Wilcoxon matched-pairs signed rank test.

### Data and code accessibility

All data used for the analyses will be made available upon request to the corresponding author. All codes used to preprocess LFPs, train factor models, make EMG-informed cluster-based sleep labels, and smooth labels are available online at https://github.com/carlson-lab/lpne. Additionally upon acceptance, this code will be input into the Duke University Research Data Repository, providing a permanent DOI and guaranteeing access for at least 30 years.

## Results

### Development and validation of brain-based sleep classifiers

To characterize oscillatory dynamics across the sleep-wake cycle, we implanted 9 C57BL/6J male mice with wires in eight cortical and subcortical brain regions along with an EMG wire in the trapezius muscle. Mice had electrode wires targeting the cingulate cortex (CxCg), prelimbic cortex (CxPrL), infralimbic cortex (CxIL), nucleus accumbens (core and shell grouped together; NAc), amygdala (basolateral and central grouped together; Amy), medial dorsal thalamus (ThalMD), ventral hippocampus (HippV), and ventral tegmental area (VTA; Fig. 1*A*; Mague et al., 2022). These regions were chosen given their well-established role in regulating emotional behavior under normal and pathological conditions (Nestler et al., 2002; Hultman et al., 2016, 2018) and are routinely targeted with our microwire electrode arrays (Mague et al., 2022; Hughes et al., 2024). In order to determine the ground truth sleep-wake state label for each time window, we hand-scored a random 1 h recording for wake, NREM, and REM using EMG power and CxPrL power based on known characteristics of these states (Keene and Duboue, 2018). We chose to bin the data into 2 s windows to reduce the number of time windows with multiple ground truth sleep labels while maintaining reliable spectral estimates. We established our accuracy benchmark based on interrater reliability from three raters on representative data used in this study, where all datapoints were classified. The average Cohen’s kappa over four 1 h recordings and pairwise comparison of three independent raters was 0.82; range, 0.80–0.89; and average agreement, 93.4% wake, 91.6% NREM, 63.1% REM. These results were in line with agreement typically reported between hand-scoring by different experts (Lee et al., 2022).

**Figure 2.**
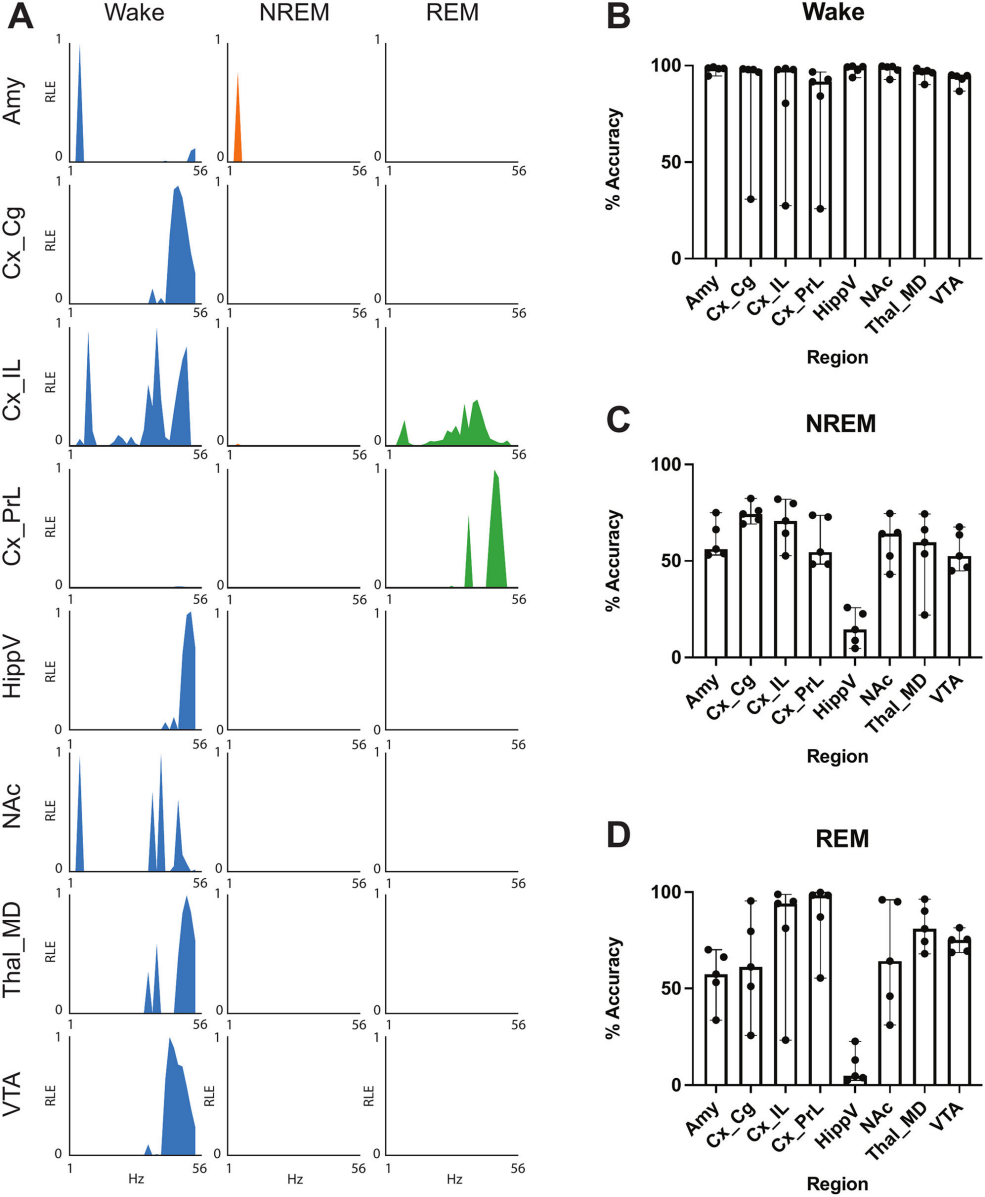
Single-region classifiers for sleep states. ***A***, Relative spectral energy (RLE) across frequencies from 1 to 56 Hz for each single-region model (rows) for each sleep-wake state (columns). ***B–D***, Single-region classifier accuracy for wake (***B***), NREM (***C***), and REM (***D***) states in hold-out male mice (*n* = 5). Data shown as median ± 95% CI.

We then generated EMG-informed labels using traditional cortical LFP power and trapezius EMG power recorded over 24 h per mouse by manual cluster identification, after validating these manual cluster-based labels with ground truth hand-scored labels (Fig. 1*D*). Windows with features that did not clearly belong to a given cluster were left unclassified. EMG-informed labels were 95.5% in agreement for wake, 99.0% in agreement for NREM, and 91.6% in agreement for REM with the hand-scored labels from a randomly sampled hour of data from seven mice. The human rater could not classify 12.6% of the data, whereas 17.3% of the data were unclassifiable using the EMG-informed method (*n* = 4 male mice, 1 randomly sampled hour of recording data each). We proceeded with using EMG-informed labels for all training mice for further classifier training, since classification accuracy met the benchmark goal based on interrater reliability (Lee et al., 2022). First, we wanted to determine the ability of each emotion-regulating brain region to decode sleep on its own. We developed SAE classifiers to predict sleep-wake states. Each classifier was trained solely using spectral power from 1 to 55 Hz from one of the brain regions (CxCg, CxPrL, CxIL, NAc, Amy, ThalMD, HippV, and VTA; Fig. 2*A*). Classifiers for each of the brain regions examined could predict sleep-wake states above chance levels, based on 33% balanced accuracy being random chance (average wake accuracy, 73.6%; average NREM accuracy, 85.5%; average REM accuracy, 70.6%; *p* ≤ 0.027 for each combination of sleep-wake state and region, one-sided Wilcoxon signed rank test comparing observed accuracy to 33%, *n* = 9 based on nested cross-validation, data not shown). Power features utilized for classification were inconsistent with known state patterns such as high delta power in NREM (Fig. 2*A*). We next tested accuracy of the single-region classifier to predict sleep-wake states in hold-out subjects and found high accuracy in wake but lower accuracy in NREM and REM (median wake accuracy, 97.9%; median NREM accuracy, 57.9%; median REM accuracy, 69.7%; *p* = 0.065 for each combination of sleep-wake state and region, one-sided Wilcoxon signed rank test comparing observed accuracy to 33%, *n* = 5; Fig. 2*B–D*). The single-region model trained on CxIL had the highest balanced accuracy (76.3%) whereas HippV had the lowest balanced accuracy (40.8%).

Yet, as we failed to meet a goal of attaining 80% average balanced accuracy across all sleep-wake states with single-region classifiers in hold-out mice, we hypothesized that the integration of information from all recorded sites would outperform single-region sleep state prediction, as we had previously observed for identification of an aggressive brain state (Grossman et al., 2024). To train a network classifier to predict sleep-wake states independently of EMG, we used the same ground truth labels implemented for the single-region classifiers and calculated power and synchrony features for frequencies up through 55 Hz from each of the recorded brain regions (see Materials and Methods for more detail). We trained a SAE model to predict ground truth sleep labels based on LFP power and synchrony features (Talbot et al., 2023). Critically, this approach simultaneously reconstructs data and can predict labels using a small number of latent dimensions. We found that the model had cross-validated (within-subject) accuracies of 87.2% for wake, 88.4% for NREM, and 79.7% for REM, compared with the “ground truth” EMG-based approach (Fig. 1*E*). Thus, the average recall of the classifier across all three sleep-wake states yielded an average balanced accuracy of 84.9%. The network classifier’s average balanced accuracy was significantly better than most single-region classifiers except for Amy and NAc in the training mice (*p* = 0.248 and *p* = 0.082, for Amy and NAc, respectively; one-sided Wilcoxon rank sum tests, *n* = 9) and significantly better than both of these single-region classifiers in the hold-out dataset (*p* = 0.011 and *p* = 0.0342 for Amy and NAc, respectively; *p* = 0.014 for main effect of two-way ANOVA, followed by multiple-comparisons test with Dunnett’s correction for multiple hypothesis testing).

**Figure 3.**
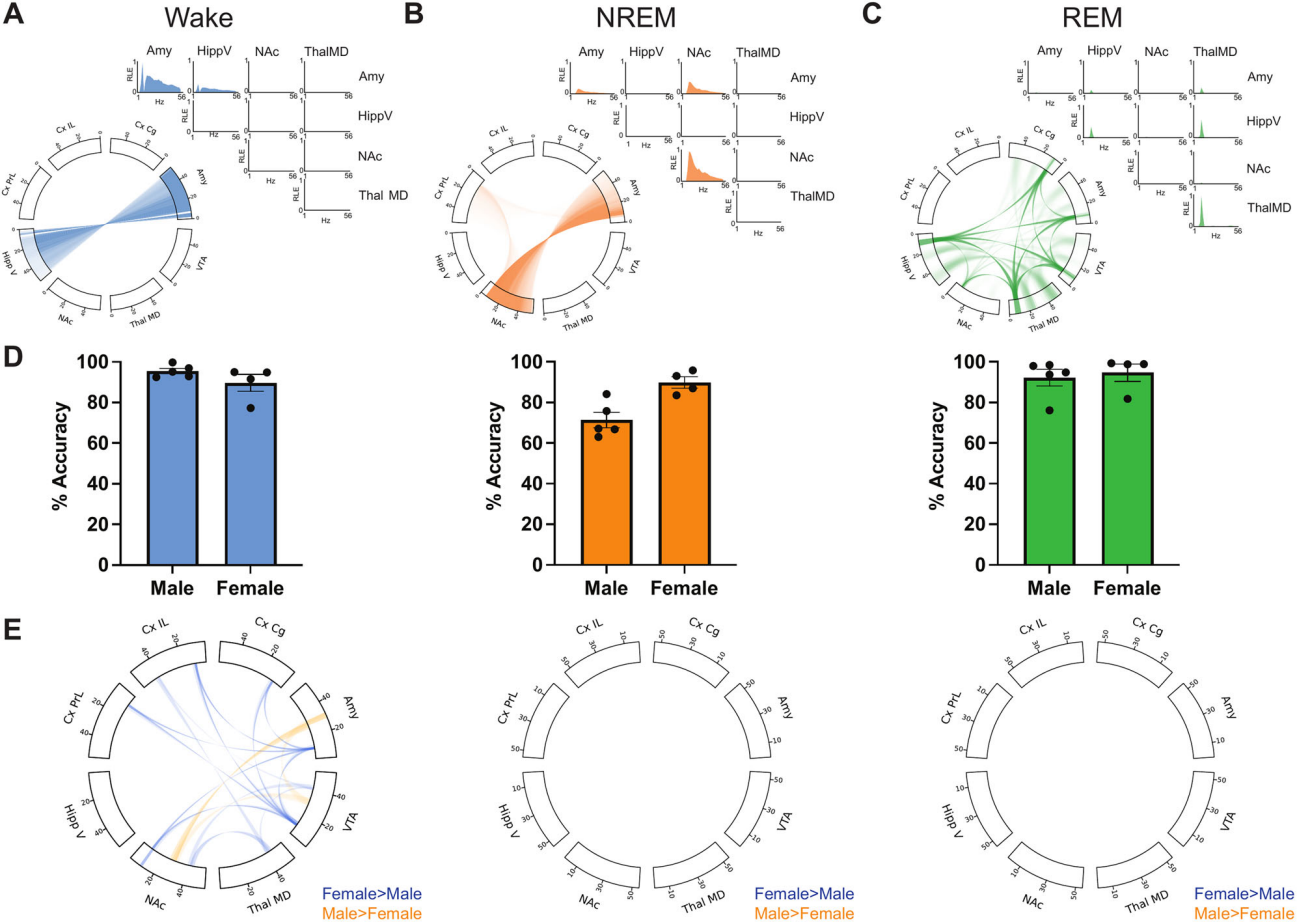
Electome network classifiers for sleep-wake states generalize between sexes. ***A–C***, Chord plots show power features from 0 to 55 Hz along the outer rim for each brain region and connections between brain regions via connecting lines between brain regions in a frequency-dependent manner. RLE plots of power (on the diagonal) and synchrony (on the off-diagonal) across 1–56 Hz in frequency are plotted for a subset of regions: Amy, HippV, NAc, and ThalMD for each sleep-wake state with (***A***) wake, (***B***) NREM, and (***C***) REM. ***D***, Average classifier accuracy in hold-out male and female mice across state (blue, wake; orange, NREM; green, REM). Mean ± SEM. ***E***, Power and synchrony differences between male and female mice across sleep-wake states. Values increased in females in comparison with males are in dark blue and values increased in males in comparison with females are in orange. Significant sex differences were seen only in the wake state (*p* = 0.0497, corrected headline harmonic mean *p* value across 1,856 two-sided independent *t* tests). Display threshold based on features that reach unadjusted significance at *α* = 0.01. *N* = 5 males/4 females.

We next determined the classifiers’ agreement with the EMG-informed labels in five new male hold-out mice. We found 86% ± 2% balanced accuracy (95% ± 2% agreement for wake, 71% ± 4% agreement for NREM, and 92% ± 4% agreement for REM; all values are mean ± SEM; Fig. 3*D*, male). In comparison with the mice used in cross-validation, there was no significant difference in balanced accuracy (*p* > 0.999, two-tailed Mann–Whitney *U* test, *n* = 5–9). We next wanted to compare the discrimination of the multiregion and single-region classifiers in hold-out subjects. We hypothesized that the single-region classifiers would not decode as well as the multiregion classifier, given our prior work (Mague et al., 2022; Hughes et al., 2024). The Amy and NAc models (that performed similar to the network classifier during cross-validation) were significantly less accurate in hold-out mice (*p* = 0.0312 for both Amy and NAc compared with the network classifier, using one-sided Wilcoxon rank sum tests, *n* = 5). Although not as accurate as the multiregion classifier, the Amy and NAc classifiers had 71 and 75% balanced accuracy, respectively. Thus, the network-based sleep-wake state classifier, and not the single-region classifiers, had similar or improved balanced accuracy in the hold-out mice in comparison with the cross-validation analysis in the training mice. Due to this improved generalization, the network-based classifier was used for all following analyses.

Next, we sought to test whether the network classifiers generalized to female mice. Female mice, in which both multisite brain and EMG were recorded, exhibited high accuracy across the network-based sleep-wake state classifiers (90 ± 4% accuracy for wake, 90 ± 3% for NREM, and 95 ± 4% for REM, 91.6% average balanced accuracy, *n* = 4; all values are mean ± SEM; Fig. 3*D*, female) with statistically indistinguishable balanced accuracies from the cross-validated male accuracies (*p* = 0.414, two-sided Mann–Whitney *U* test, *n* = 4–9). Thus, the network-based classifier identified for sleep-wake states in male mice generalized to female mice.

### Power and synchrony profiles of distributed brain activity in wake, NREM, and REM

**Figure 4.**
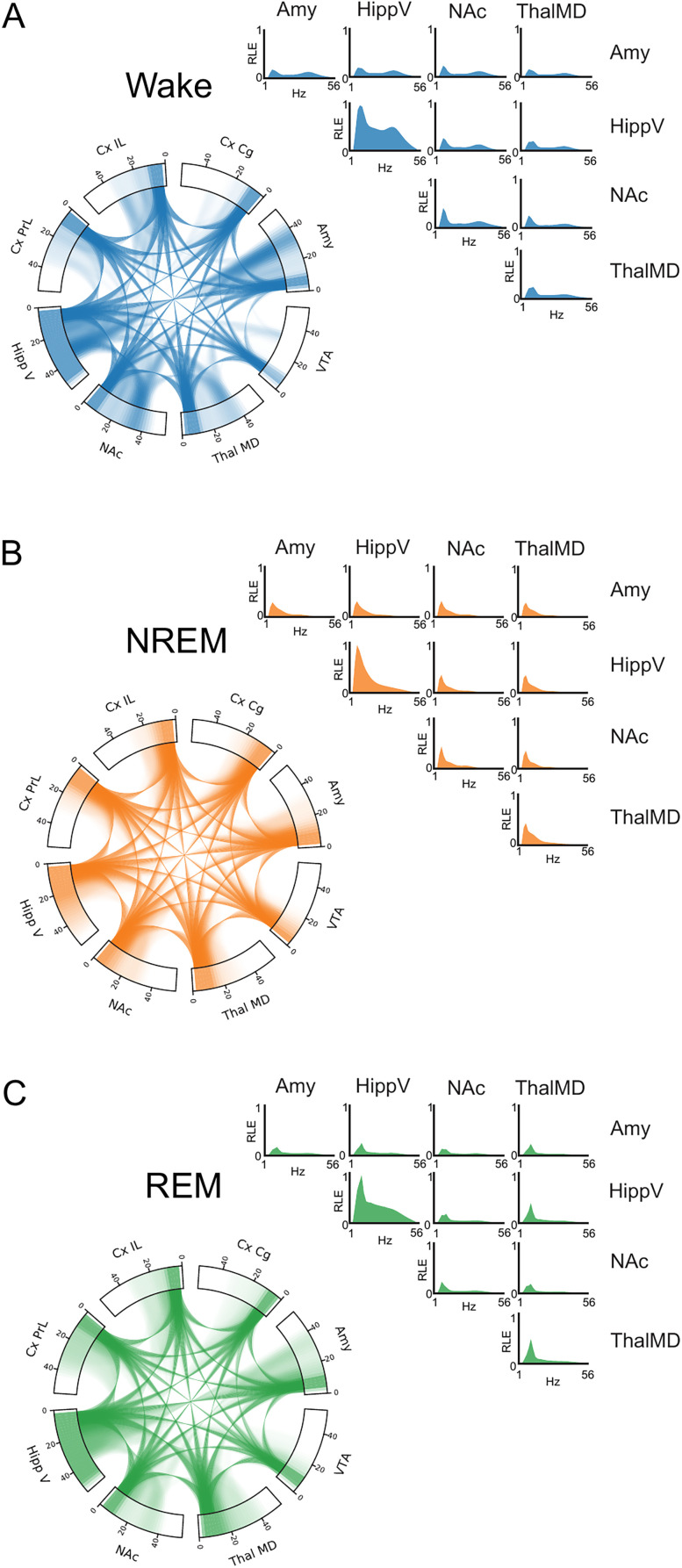
Average brain activity during wake, NREM, and REM. Chord plots showing power features from 0 to 55 Hz along the outer rim for each brain region and connections between brain regions via connecting lines between brain regions in a frequency-dependent manner. RLE plots of power (on the diagonal) and synchrony (on the off-diagonal) are plotted for Amy, HippV, NAc, and ThalMD for (***A***) wake, (***B***) NREM, and (***C***) REM. Unthresholded average feature strengths plotted. *N* = 9 male mice.

We then sought to describe the characteristic brain activity for each sleep-wake state across the monitored regions. To examine the brain activity during each state, we averaged power and synchrony across 24 h for all timepoints identified for each state based on our network classifier. REM average power and synchrony features were more similar to wake (Fig. 4*A*) than NREM features (0.94 vs 0.84 cosine similarity), a difference that held across all 9 mice used to train the classifier. NREM brain activity was dominantly composed of large delta (1–4 Hz) activity without higher frequency activity (Fig. 4*B*). Wake and REM also were composed of delta power across all regions and exhibited higher frequency activity (Fig. 4*A*,*C*). We observed broad-spectrum activity (2–52 Hz) in HippV during wake and REM (Fig. 4*A*,*C*). Higher frequency (26–40 Hz) power and synchrony was also observed in Amy, IL, PrL, NAc, and ThalMD during wake and REM, albeit more distinct during wake (Fig. 4*A*,*C*). The average brain features from each state were consistent with the EEG-based brain oscillations that define sleep-wake states (Kohtoh et al., 2008).

To further disambiguate the sleep-wake states, we next examined the components of the supervised electome network for each of them. In contrast to the average brain activity in each state, the SAE models learn features that best discriminate the states from one another. According to the model, wake was associated with increased power in Amy from 4–8 and 12–55 Hz as well as synchrony between Amy and HippV in the same ranges compared with the other two states (Fig. 3*A*). NREM was associated with increased 10–55 Hz activity in NAc and to a lesser extent to Amy as well as synchrony between NAc, CxPrL, and Amy (Fig. 3*B*). REM was associated with increased 6–12 Hz activity as well as 18–26 and 42–50 Hz activity in ThalMD and HippV and to a lesser extent in VTA, Amy, and CxCg as well as synchrony among HippV, NAc, ThalMD, VTA, Amy, and Cg (Fig. 3*C*). This finding extends widespread synchronous activity in sleep to subcortical regions including NAc, VTA, and Amy. Moreover, we establish the widespread synchronous activity profiles across brain regions for emotional regulation that are observed during distinct sleep states.

### Sex comparison shows no significant difference in brain dynamics during sleep

Even though the classifier generalized from male to female mice, it is still possible that male and female mice exhibit distinct oscillatory features during sleep that are separate from the features used by the classifier. We tested this hypothesis by examining the average power and synchrony features within the wake, NREM, or REM epochs identified by the network classifier during 24 h recordings of 13 male mice and 11 female mice. While NREM and REM had no significant differences between males and females (*p* = 0.389 for NREM and *p* = 0.403 for REM, corrected headline harmonic mean *p* value across 1,856 two-sided independent *t* tests), wake brain activity pattern was significantly different (*p* = 0.0497, corrected headline harmonic mean *p* value across 1,856 two-sided independent *t* tests). Female mice on average exhibited increased synchrony between Amy and VTA from 38 to 52 Hz, while male mice on average had increased synchrony in the same projection from 30 to 34 Hz during wake (Fig. 3*E*). Additionally, female mice had increased 10–12 Hz synchrony between NAc and VTA and between Amy and VTA (Fig. 3*E*). Thus, NREM and REM brain activity is not statistically different between males and females, whereas wake has significant sex differences in synchrony in subcortical brain structures.

### Trazodone and diazepam alter sleep architecture and brain dynamics across sleep-wake states

We next explored the widespread electrophysiological properties induced by two pharmacological agents commonly used to promote sleep—trazodone and diazepam. Drug concentrations were based on previous literature examining these drugs in mice (Kopp et al., 2003; de Oliveira et al., 2022). Trazodone decreases sleep latency and increases sleep duration with improved efficiency of sleep and increased time spent in NREM, in addition to serving as an antidepressant (Jaffer et al., 2017). The anxiolytic diazepam decreases sleep latency, increases sleep duration, and paradoxically also decreases the amount of REM (Holbrook et al., 2001). Thus, we examined the effects of these mood-modulating agents on state-dependent brain dynamics. Here, we used a crossover design where trazodone, diazepam, or vehicle control was administered acutely.

**Figure 5.**
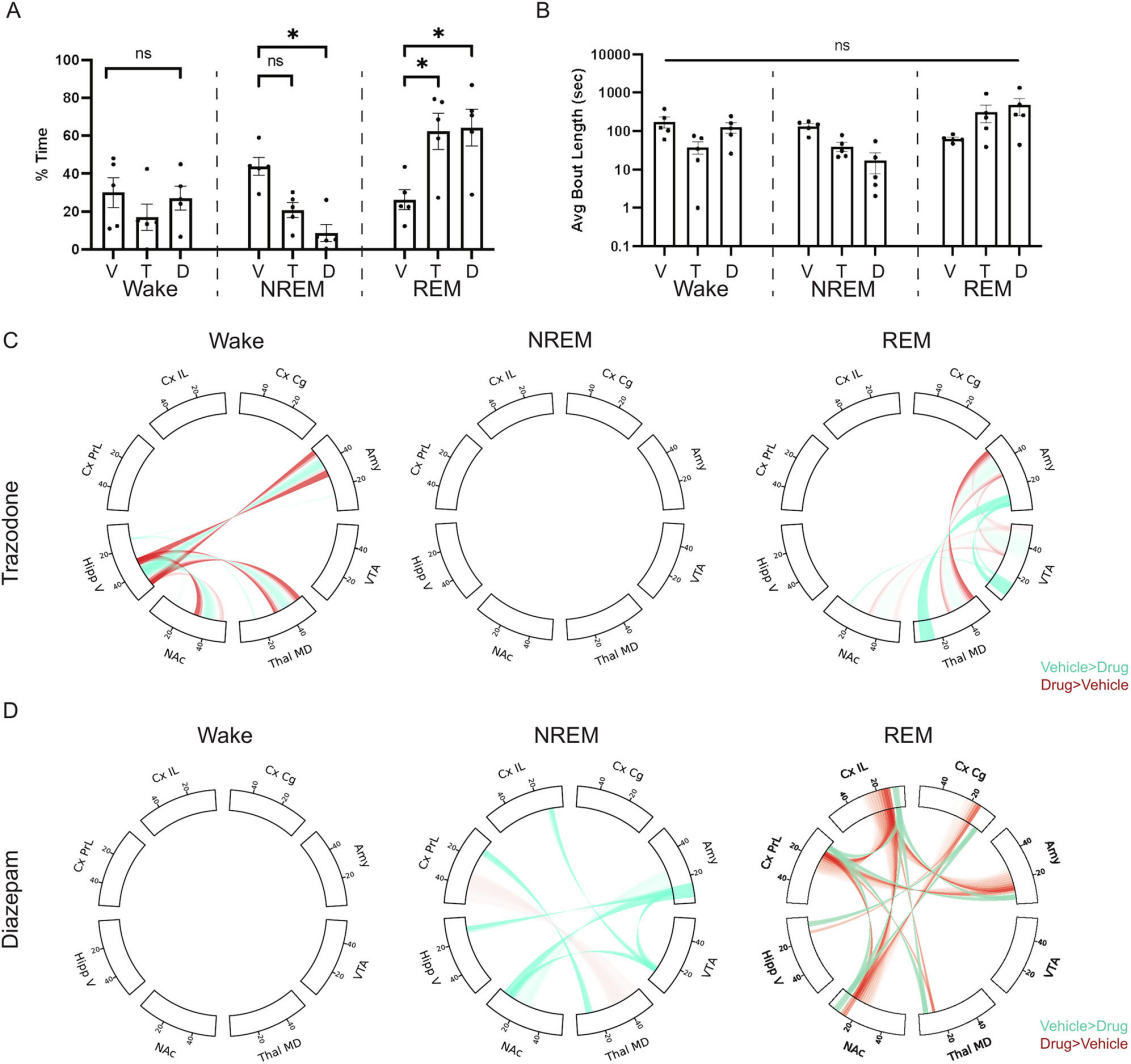
Brain-wide response to acute sleep medication. ***A***, Trazodone (T) and diazepam (D) alter overall time in NREM and REM (but not wake) states during the 4 h after acute administration in comparison with vehicle (V) (two-way RM-ANOVA, *F*_(2.016,8.064)_ = 6.064 for drug by state interaction *p* = 0.025). ***B***, No change in average bout length after acute administration of trazodone (T) or diazepam (D) in comparison with vehicle (V; two-way RM-ANOVA, *F*_(1.614,6.458)_ = 4.068 for drug by state interaction *p* = 0.078). ***C***, ***D***, Significant power and synchrony differences in the 4 h period after acute trazadone (C) or diazepam (D) based on sleep network classifier-state identification. Power and synchrony features that are increased in vehicle in comparison with drug are shown in teal; increased in drug in comparison with vehicle are shown in red. Threshold based on features that reach unadjusted significance at *α* = 0.01. *N* = 5 male mice. *p < 0.05, **p < 0.01.

To identify acute effects of trazodone and diazepam on sleep architecture, we first quantified sleep-wake states for the 4 h after drug administration using the network-based classifiers. We observed significant alterations to sleep state duration (drug by state: *F*_(2.016,8.064)_ = 6.064, two-way RM-ANOVA, *p* = 0.025). Indeed, diazepam induced significant decreases in NREM time (*p* = 0.01, Dunnett’s multiple-comparison test). Trazodone tended to decrease NREM time as well, though this change did not reach statistical significance (*p* = 0.09, Dunnett’s multiple-comparison test). Both agents increased REM time relative to vehicle administration (trazodone *p* = 0.05, diazepam *p* = 0.02, Dunnett’s multiple-comparison test), with no significant change in wake time (trazodone *p* = 0.29, diazepam *p* = 0.89, Dunnett’s multiple-comparison test; Fig. 5*A*). There were no significant changes in average bout length across sleep-wake states after treatment with both drugs compared with vehicle (*F*_(1.614,6.458)_ = 4.068, two-way RM-ANOVA, *p* = 0.078; Fig. 5*B*). However, there was a wide variation in bout length for REM after both trazodone and diazepam relative to vehicle (trazodone *p* < 0.01, diazepam *p* < 0.01, *F* test to compare variances; Fig. 5*B*).

We next sought to examine the brain dynamics across recording sites to examine acute effects of trazodone and diazepam during each sleep-wake state. Trazodone changed brain activity significantly during both wake and REM epochs (*p* = 0.0417 and 0.0113, respectively, harmonic *p* value), and brain activity changes trended toward significance for NREM (*p* = 0.0662, harmonic *p* value). During wake, trazodone caused increased synchrony between Amy/ThalMD/NAc and HippV in comparison with control ∼30 and 50 Hz but decreased ∼40 Hz (Fig. 5*C*). During REM, trazodone increased synchrony ∼30 and 50 Hz and decreased synchrony ∼40 Hz between ThalMD and Amy, NAc and VTA, and VTA and Amy. Additionally, there was decreased power 6–16 Hz in ThalMD and VTA as well as decreased synchrony in that same band between ThalMD and Amy (Fig. 5*C*). Diazepam significantly altered brain activity during both NREM (*p* = 0.0112, harmonic *p* value) and REM (*p* = 0.0020, harmonic *p* value) but not wake (*p* = 0.130, Holm–Šídák's multiple-comparisons test). Diazepam in comparison with vehicle decreased 8–16 Hz synchrony in CxIL and VTA, CxPrL and ThalMD, HippV and Amy, NAc and Amy, and NAc and VTA, and a broad frequency (24–56 Hz) increase in synchrony was observed between CxPrL and ThalMD after drug administration during NREM (Fig. 5*D*). During REM, diazepam increased ∼14 Hz synchrony between CxIL and CxPrL, CxPrL/CxIL and NAc, CxPrL/CxIL and Amy, and CxCg and NAc. There was decreased 6–10 Hz synchrony among the same projections relative to vehicle administration. Additionally, there was increased power ∼14 Hz and decreased power ∼6–10 Hz in CxIL and NAc in comparison with vehicle (Fig. 5*D*). Thus, trazodone and diazepam increase sleep and elicit differences in brain dynamics during sleep-wake states relative to sleep brain activity observed with vehicle administration.

### Chronic circadian disruption alters sleep architecture without changing sleep brain dynamics

**Figure 6.**
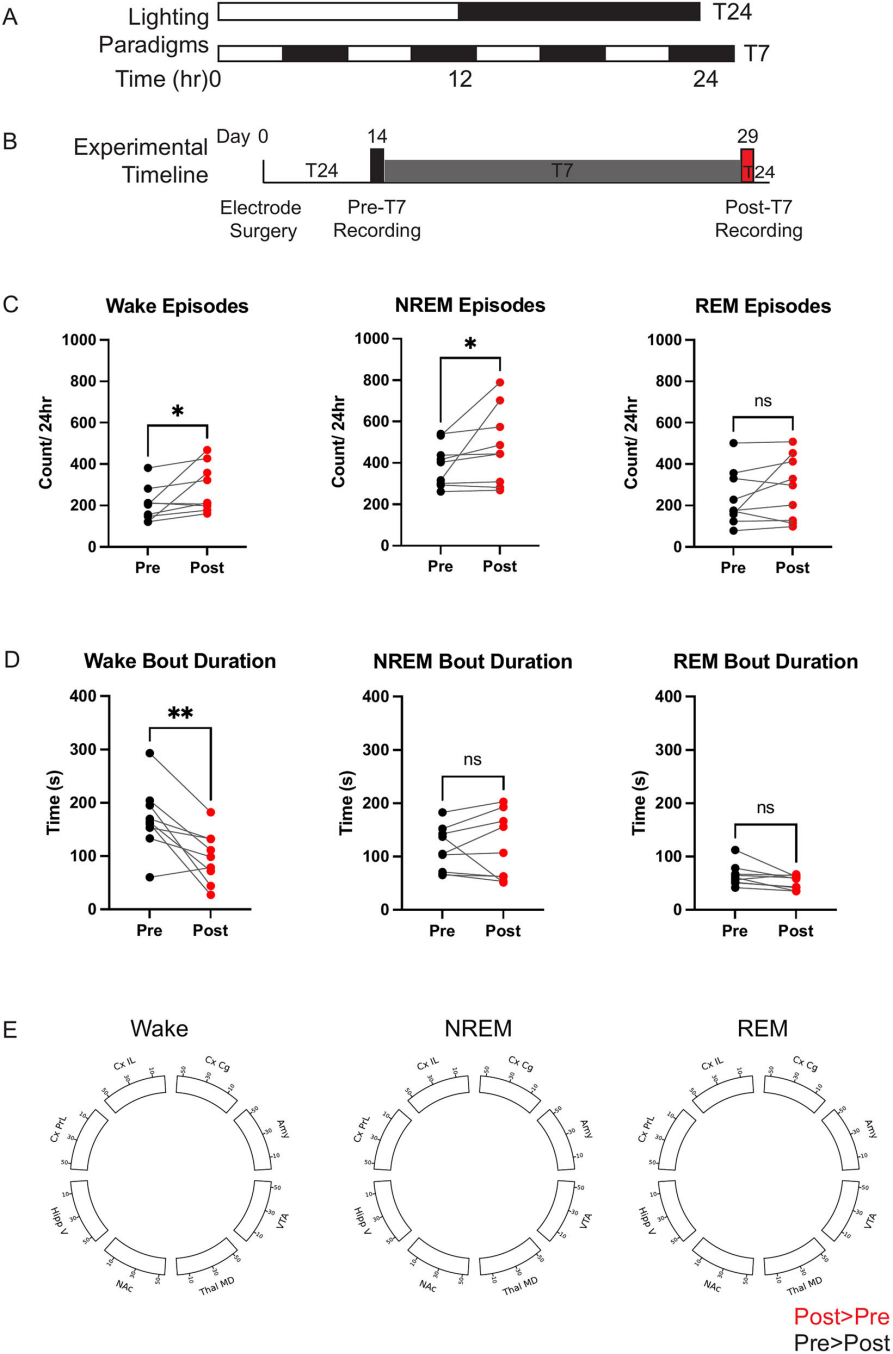
Chronic circadian disruption stress alters sleep architecture with no change to average state brain activity. ***A***, Scheme of light cycle conditions used during recording. T24 (12 h on/12 h off) was used during all pre- and postaberrant light times. T7 (3.5 h on/3.5 h off) was used for circadian disruption. ***B***, Timeline of prestress (black) and poststress (red) experimental recordings for the chronic stress protocol. ***C***, Increase in the number of episodes of wake and NREM (but not REM) after 14 d of T7 light stress. ***D***, Significant decrease in bout duration of wake and marginally significant decrease in REM (but not NREM) bout duration following 14 d of T7 light stress. ***E***, Lack of any significant changes in averaged brain activity in each state following chronic stress. If significant, increased power or synchrony after stress would be shown in red, and decreased power or synchrony after stress would be shown in black. One-tailed Wilcoxon matched-pairs signed rank test. *N* = 9 male mice. *p < 0.05, **p < 0.01.

Finally, we examined sleep in a chronic stress-induced, depressive-like model generated by aberrant light exposure to test if an emotion perturbation paradigm would change sleep quantity or content (Fig. 6*A*; based on LeGates et al., 2012). Behavioral assessment of mice exposed to an abbreviated light/dark cycle (T7) for 14 d has been reported to resemble a depressive-like phenotype, with decreased sucrose preference and increased immobility time during the forced swim test (LeGates et al., 2012).

We first examined the quantity of sleep by determining the overall percentage of time spent in each sleep-wake state over the course of 24 h, before and after 2 weeks of light stress (Fig. 6*B*). Similar to prior reports, we observed no change to overall amounts of wake, NREM, or REM over the course of 24 h (LeGates et al., 2012). However, after stress exposure, mice exhibited increased sleep fragmentation as indicated by increased number of episodes of wake and NREM (wake *p* = 0.0195, NREM *p* = 0.0273, Wilcoxon matched-pairs signed rank test; Fig. 6*C*) and decreased bout length for wake (wake *p* = 0.0078, Wilcoxon matched-pairs signed rank test; Fig. 6*D*). Mice also tended to show a decrease in the bout length for REM, though these differences did not reach statistical significance (REM *p* = 0.0547, Wilcoxon matched-pairs signed rank test; Fig. 6*D*). Then we examined brain dynamics during each sleep/wake state by comparing power and synchrony across all recorded brain regions for each sleep classifier-based label before and after 14 d of T7. Averaging over the 24 h pre- versus poststress periods, we observed no significant T7-induced changes in power or synchrony across the brain (wake *p* = 0.204, NREM *p* = 0.261, REM *p* = 0.361, corrected headline harmonic mean *p* value across 1,856 two-sided independent *t* tests). Thus, chronic aberrant light cycle disruption induced sleep fragmentation abnormalities in mice, with no difference in average brain dynamics of emotion-regulating regions during sleep states.

## Discussion

Here, we used a machine learning-based electome classifier to identify patterns of power and synchrony across emotion regulation brain region that distinguish wake, NREM, and REM states in mice, independent of electromyographic recordings. Oscillatory power in any one region, originally chosen due to their implication in emotional regulation or generation, could independently predict sleep-wake state at a level significantly higher than chance. We then developed classifiers using power and synchrony from all recorded brain regions and identified an electome model composed of a supervised component for each sleep-wake state, indicating that there are unique, stable patterns of brain activity across the observed regions during each sleep-wake state. This multiregion classifier performed significantly better than all single-region classifiers and was conserved between sexes. We then examined brain dynamic perturbations induced by two common psychotropic drugs known to modulate mood and sleep in humans and rodents. Both drug classes induced prominent differences in brain dynamics across sleep-wake states. Finally, we used the network classifier to identify disrupted sleep architecture in a chronic circadian stress-induced model of depression-like behavioral dysfunction. We found that chronic circadian stress increases sleep fragmentation, but the average brain dynamics of each sleep state are indistinguishable in the pre- and poststressed conditions.

A key methodological advance from our study is a novel strategy for sleep-wake classification from multiregion electrophysiological recordings. Sleep-wake classification using hand-scoring by trained experts, which remains common in the field, not only requires reliable EMG recordings but is also time and labor intensive. Here we developed accurate classification of sleep-wake states utilizing EMG-free network classifiers from LFP recordings of several emotion regulation brain regions. Mouse models of psychiatric disease are particularly susceptible to conditions that are incompatible with stable, long-term EMG recordings. Longitudinal studies examining sleep require EMG for the duration of the study; however, the average duration of a quality EMG signal was 1 month postimplantation, making any aging study or long-term longitudinal study impossible (such as in chronic stress paradigms; Antoniuk et al., 2019). Additionally, many psychiatric disease models are incompatible with quality EMG for various reasons, including increased grooming (e.g., autism and obsessive-compulsive disorder; Welch et al., 2007; Wang et al., 2016), physical exertion (e.g., aggression; Golden et al., 2019), and excessive handling (e.g., restraint stress; Mao et al., 2022) or social defeat (Berton et al., 2006). Similar to their human counterparts, traditional methods of sleep-wake state identification are not feasible in subjects that are unable to keep a muscle probe attached, which led our group and others to try to develop EMG-free classification approaches (Estrada et al., 2006; Ulrich et al., 2015; Bastianini et al., 2017; Kloefkorn et al., 2020; Zhang et al., 2022). However, these methods require time-consuming manual annotation of alternative signals such as whole-body plethysmography (Bastianini et al., 2017; Kloefkorn et al., 2020) or electrical field sensors (Zhang et al., 2022). The development of non-EMG-based classifiers of sleep-wake states allowed for tractable, long-term determination of sleep and wake in rodent models of emotional perturbation, opening the ability to examine mechanisms of sleep alterations without tedious manual annotations. In the EMG-free classifiers, we observed recapitulation of many of the known neurophysiological signatures for each of the states, such as low-amplitude, mixed-frequency power and synchrony in wake versus high-amplitude, low-frequency power and synchrony in NREM (Keene and Duboue, 2018). In terms of features most important for discriminating the states, Amy was implicated in both the wake and NREM classifiers with unique signatures of synchrony with the HippV and NAc, respectively. This suggests that Amy is a key brain region in sleep-wake state identification in comparison with the other regions monitored in this study. This adds to the growing literature showing the importance of Amy in sleep, where recent evidence showed that amygdala—in particular the basolateral amygdala—initiated the REM state (Hasegawa et al., 2022). While outside the scope of this study, future work manipulating the activity of amygdalar circuits could be undertaken to see if this alters sleep-wake states and emotional functioning.

We have previously used this approach to decode emotional electrical functional connectomes—or electomes—from brain activity recorded in mice (Mague et al., 2022; Grossman et al., 2024; Hughes et al., 2024). However, these electomes required much larger training datasets (2–5-fold more mice) to identify stable electome representations. In contrast, our ability to decode sleep-wake states here with just nine training mice is likely indicative of the greater consistency of sleep-wake brain dynamics relative to emotional brain dynamics between subjects, the very distinct electrophysiological signatures of sleep versus wake, and the ability to generate more accurate sleep-wake behavioral labels in comparison with labeling emotional states. Due to these robust and relatively consistent signals used to classify sleep-wake states, it is unlikely that valence- or emotion-based experiences would significantly alter these brain dynamics within our recorded sleep- and emotion regulation-related brain regions to diminish the accuracy of sleep-wake classification here.

Secondly, our study continued to shed light on the interplay between sleep encoding in brain regions associated with emotion regulation. The sequence in which mental health conditions and sleep disruption emerge is unclear, but there is evidence to support that normalizing sleep can improve emotion regulation either before or after the emergence of a psychiatric condition (Goldstein and Walker, 2014; Jagannath et al., 2017). By better understanding the relationship between sleep and emotion regulation, we could generate better treatments to promote overall health. It will be important to conduct longitudinal studies over the course of disease progression to disentangle the relationship between sleep disruption and mental health symptom severity. Additionally, several classes of compounds commonly used to treat emotional disturbance are known to have sleep-promoting effects. The neural dynamics underlying these effects have not been previously examined. However, here we show that trazodone and diazepam increased the likelihood of brain activity being classified as REM after acute administration and that the average brain dynamics classified during REM are altered differently by the two compounds. It is unclear if this altered REM brain state was as beneficial as unmedicated REM-like sleep for an organism. By developing drugs that promote sleep with normal sleep brain dynamics, we could disentangle sleep benefits from the medications’ other effects.

Our network classifiers allowed us to investigate the sleep architecture in a mouse model of psychiatric disease. Indeed, we detected increased sleep fragmentation in a model of chronic stress-induced behavioral adaptation generated by circadian disruption. Consistent with previous reports, the overall sleep quantity is unaffected (Altimus et al., 2008; LeGates et al., 2012). However, further analysis revealed the fragmentation phenotype, in which state bout lengths were decreased. Whether the observed increase in sleep fragmentation represents a stress-related effect or a compensatory mechanism is an intriguing hypothesis to explore in future experiments. Additionally, since the brain dynamics during sleep were indistinguishable before and after the depression-like manipulation, normal sleep-inducing treatments may be beneficial at ameliorating sleep differences in those with depression. Interestingly, sleep reactivity, or the degree to which an individual’s sleep deteriorates when stressed, is correlated to the individual’s propensity for developing both pathological sleep disorders as well as other psychological disorders (Kalmbach et al., 2018), which highlights the translational value of studying mechanisms of stress-induced sleep disruption in mice.

Although average brain dynamics were unaltered during sleep-wake states in the model of chronic stress, other brain dynamic changes may exist. Namely, sleep state transitions are disrupted in depression (Tsuno et al., 2005; Franzen and Buysse, 2008; Riemann et al., 2020). Indeed, we observed increased sleep fragmentation after chronic stress (Fig. 6*C*). Now that our approach can rapidly classify the sleep-wake states, future research could dissect the neural features that underlie state transitions by examining the timepoints immediately preceding state change. Identifying the processes that regulate the transitions among sleep states could reveal new avenues for intervention for sleep transition disruptions.

Trazadone and diazepam are reported to increase sleep in people and rodents. Specifically, studies have reported an increase in NREM and REM sleep following diazepam administration and an increase in NREM following trazadone administration (McKillop et al., 2021; Arai and Kent, 2025). These studies rely on sleep-wake state classification based on EEG and EMG, and they show that EEG power spectra are altered during NREM and REM but less so during wake. Because the multiregion classifier lacks muscle tone information, we are solely reliant on the brain activity patterns to discriminate states. Thus, our determination of sleep-wake state is based on which classifier is most similar to the observed brain activity for a particular state. Thus, we believe that trazodone and diazepam induce a REM-like brain state after acute administration. Diazepam induced a focal increase in power during REM in both our study (Fig. 5*D*) and previous reports (McKillop et al., 2021), albeit at different frequencies. We observed no significant cortical alterations in response to trazadone (Fig. 5*C*) in contrast to reports using EEG (Arai and Kent, 2025).

Our study did have several important limitations. First, while we were able to achieve sleep-wake state classification accuracy on par with other published approaches (Kloefkorn et al., 2020; Lee et al., 2022; Zhang et al., 2022), there was still some disagreement in labels between machine learning classifiers versus semi-automated or fully hand-scored labels and between different human raters. It has been difficult to achieve >85% accuracy in rodent studies as compared with human sleep studies due to several reasons: (1) lack of consensus for criteria for rodent sleep-wake states; (2) most researchers, including those in this study, collapsing NREM into one state that is synonymous with slow-wave sleep in rodent studies, as opposed to the four substates of NREM comprising both light and slow-wave sleep in humans; and (3) the short duration of rodent sleep states, which results in greater numbers of sleep-wake transition epochs that are often difficult to classify (Rayan et al., 2024). Instead of using a supervised approach, future studies could use an unsupervised model training approach to classify states based on observed neurophysiological features, which may further refine our understanding of mouse sleep-wake substates and transitions. Second, our study focused on how sleep-wake states are encoded in brain regions involved in emotion regulation due to the co-occurrence and bidirectional interplay of challenges in emotion regulation and sleep across many psychiatric conditions. We did not know a priori the contribution of the monitored brain regions to sleep encoding. Of note, CxIL was only a weak contributor to any of the three sleep-wake state classifiers (Fig. 3*A–C*) even though prior work has shown its modulation during sleep states (Hong et al., 2024). As such, our prior attempts to identify negative control regions—regions that do not encode any state of interest in some capacity—in network identification have failed, likely due the widespread changes in neural activity that occur across brain states (Grossman et al., 2024). Other brain regions that were not recorded in this study are likely to show unique patterns of activity across sleep-wake states. Thus, we argue that including such brain regions would likely improve accuracy in sleep-wake classification, even if they do not directly modulate sleep. Thus, the classic framework underlying the identification and verification of negative control brain regions is unlikely to apply in this context.

Future work could extend this study by (1) recording from other brain regions to assess their contribution to state identification and to perturbation with emotional manipulations, which could identify selective impacts on power or synchrony in these additional regions; (2) recording sleep in a communal setting to not induce isolation stress; and (3) examining chronic stress manipulations that produce alterations to brain dynamics that are not captured by aggregate averaged state activity. Finally, many new observations were made about the impacts of different manipulations on power and synchrony across brain regions during sleep-wake states. The mechanistic relevance of these specific features used to delineate sleep-wake states to sleep as a biological process are not yet known, as few studies have recorded from these regions simultaneously during sleep-related experiments. Future work could examine how altering power or synchrony in specific circuits impacts sleep architecture or emotion regulation behaviors.

### Conclusion

In conclusion, we sought to determine the utility of emotion-associated regions for encoding sleep, as well as the impacts of psychopharmacological and stress manipulations on their dynamics during sleep states. We developed an accurate, high-throughput sleep-wake state classifier for brain-wide recordings in mice that can be applied across sexes and to a disease model. We validated the classifier and examined brain dynamics of emotion-associated regions during sleep states to compare sex differences, as well as the effects of emotion regulation medications and stress-induced behavioral dysregulation. Sleep is a brain-wide phenomenon that impacts regions highly implicated in emotional disruption. Thus, the encoders described here allow for further dissection of the mechanisms comodulating sleep and emotional health.

## Synthesis

Reviewing Editor: Katrina Choe, McMaster University Decisions are customarily a result of the Reviewing Editor and the peer reviewers coming together and discussing their recommendations until a consensus is reached. When revisions are invited, a fact-based synthesis statement explaining their decision and outlining what is needed to prepare a revision will be listed below. The following reviewer(s) agreed to reveal their identity: Mattia Aime, Lisa Genzel. Note: If this manuscript was transferred from JNeurosci and a decision was made to accept the manuscript without peer review, a brief statement to this effect will instead be what is listed below.

The reviewers are in agreement that the revision has addressed all concerns.

## References

[B1] Altimus CM, Guler AD, Villa KL, McNeill DS, Legates TA, Hattar S (2008) Rods-cones and melanopsin detect light and dark to modulate sleep independent of image formation. Proc Natl Acad Sci U S A 105:19998–20003. 10.1073/pnas.0808312105 19060203 PMC2596746

[B2] Antoniuk S, Bijata M, Ponimaskin E, Wlodarczyk J (2019) Chronic unpredictable mild stress for modeling depression in rodents: meta-analysis of model reliability. Neurosci Biobehav Rev 99:101–116. 10.1016/j.neubiorev.2018.12.002 30529362

[B3] Arai M, Kent BA (2025) The effects of acute trazodone administration on sleep in mice. Sleep Adv 6:zpaf031. 10.1093/sleepadvances/zpaf031 40524941 PMC12168124

[B4] Arrigoni E, Saper CB (2014) What optogenetic stimulation is telling us (and failing to tell us) about fast neurotransmitters and neuromodulators in brain circuits for wake-sleep regulation. Curr Opin Neurobiol 29:165–171. 10.1016/j.conb.2014.07.016 25064179 PMC4268002

[B5] Aserinsky E, Kleitman N (1953) Regularly occurring periods of eye motility, and concomitant phenomena, during sleep. Science 118:273–274. 10.1126/science.118.3062.273 13089671

[B6] Baglioni C, Nanovska S, Regen W, Spiegelhalder K, Feige B, Nissen C, Reynolds CF, Riemann D (2016) Sleep and mental disorders: a meta-analysis of polysomnographic research. Psychol Bull 142:969–990. 10.1037/bul0000053 27416139 PMC5110386

[B7] Bastianini S, Alvente S, Berteotti C, Lo Martire V, Silvani A, Swoap SJ, Valli A, Zoccoli G, Cohen G (2017) Accurate discrimination of the wake-sleep states of mice using non-invasive whole-body plethysmography. Sci Rep 7:41698. 10.1038/srep41698 28139776 PMC5282481

[B8] Berton O, et al. (2006) Essential role of BDNF in the mesolimbic dopamine pathway in social defeat stress. Science 311:864–868. 10.1126/science.1120972 16469931

[B9] Besedovsky L, Lange T, Haack M (2019) The sleep-immune crosstalk in health and disease. Physiol Rev 99:1325–1380. 10.1152/physrev.00010.2018 30920354 PMC6689741

[B10] Brown RE, Basheer R, McKenna JT, Strecker RE, McCarley RW (2012) Control of sleep and wakefulness. Physiol Rev 92:1087–1187. 10.1152/physrev.00032.2011 22811426 PMC3621793

[B11] Chang CH, Chen MC, Qiu MH, Lu J (2014) Ventromedial prefrontal cortex regulates depressive-like behavior and rapid eye movement sleep in the rat. Neuropharmacology 86:125–132. 10.1016/j.neuropharm.2014.07.005 25036609 PMC4188719

[B12] Chowdhury S, Matsubara T, Miyazaki T, Ono D, Fukatsu N, Abe M, Sakimura K, Sudo Y, Yamanaka A (2019) GABA neurons in the ventral tegmental area regulate non-rapid eye movement sleep in mice. Elife 8:e44928. https://doi.10.7554/eLife.44928 31159923 10.7554/eLife.44928 PMC6548506

[B13] de Oliveira P, Cella C, Locker N, Ravindran KKG, Mendis A, Wafford K, Gilmour G, Dijk DJ, Winsky-Sommerer R (2022) Improved sleep, memory, and cellular pathological features of tauopathy, including the NLRP3 inflammasome, after chronic administration of trazodone in rTg4510 mice. J Neurosci 42:3494–3509. 10.1523/JNEUROSCI.2162-21.2022 35273086 PMC9034788

[B14] Dzirasa K, Ribeiro S, Costa R, Santos LM, Lin SC, Grosmark A, Sotnikova TD, Gainetdinov RR, Caron MG, Nicolelis MA (2006) Dopaminergic control of sleep-wake states. J Neurosci 26:10577–10589. 10.1523/JNEUROSCI.1767-06.2006 17035544 PMC6674686

[B15] Estrada E, Nazeran H, Barragan J, Burk JR, Lucas EA, Behbehani K (2006) EOG and EMG: two important switches in automatic sleep stage classification. Conf Proc IEEE Eng Med Biol Soc 2006:2458–2461. 10.1109/IEMBS.2006.260075 17946514

[B16] Franzen PL, Buysse DJ (2008) Sleep disturbances and depression: risk relationships for subsequent depression and therapeutic implications. Dialogues Clin Neurosci 10:473–481. 10.31887/DCNS.2008.10.4/plfranzen 19170404 PMC3108260

[B17] Gervasoni D, Lin SC, Ribeiro S, Soares ES, Pantoja J, Nicolelis MA (2004) Global forebrain dynamics predict rat behavioral states and their transitions. J Neurosci 24:11137–11147. 10.1523/JNEUROSCI.3524-04.2004 15590930 PMC6730270

[B18] Golden SA, Jin M, Shaham Y (2019) Animal models of (or for) aggression reward, addiction, and relapse: behavior and circuits. J Neurosci 39:3996–4008. 10.1523/JNEUROSCI.0151-19.2019 30833504 PMC6529864

[B19] Goldstein AN, Walker MP (2014) The role of sleep in emotional brain function. Annu Rev Clin Psychol 10:679–708. 10.1146/annurev-clinpsy-032813-153716 24499013 PMC4286245

[B20] Grossman YS, Talbot A, Gallagher NM, Thomas GE, Fink AJ, Walder-Christensen KK, Russo SJ, Carlson DE, Dzirasa K (2024) A widespread oscillatory network encodes an aggressive internal state. bioRxiv:2022.2012.2007.519272.

[B21] Guo Y, et al. (2023) Increased connectivity of the anterior cingulate cortex is associated with the tendency to awakening during N2 sleep in patients with insomnia disorder. Sleep 46:zsac290. 10.1093/sleep/zsac290 36462192

[B22] Hasegawa E, Miyasaka A, Sakurai K, Cherasse Y, Li Y, Sakurai T (2022) Rapid eye movement sleep is initiated by basolateral amygdala dopamine signaling in mice. Science 375:994–1000. 10.1126/science.abl6618 35239361

[B23] Holbrook A, Crowther R, Lotter A, Endeshaw Y (2001) The role of benzodiazepines in the treatment of insomnia: meta-analysis of benzodiazepine use in the treatment of insomnia. J Am Geriatr Soc 49:824–826. 10.1046/j.1532-5415.2001.49161.x 11454123

[B24] Hong J, Choi K, Fuccillo MV, Chung S, Weber F (2024) Infralimbic activity during REM sleep facilitates fear extinction memory. Curr Biol 34:2247–2255.e5. 10.1016/j.cub.2024.04.018 38714199 PMC11111341

[B25] Hughes DN, et al. (2024) A widespread electrical brain network encodes anxiety in health and depressive states. bioRxiv:2024.2006.2026.600900.

[B26] Hultman R, et al. (2016) Dysregulation of prefrontal cortex-mediated slow-evolving limbic dynamics drives stress-induced emotional pathology. Neuron 91:439–452. 10.1016/j.neuron.2016.05.038 27346529 PMC4986697

[B27] Hultman R, et al. (2018) Brain-wide electrical spatiotemporal dynamics encode depression vulnerability. Cell 173:166–180.e14. 10.1016/j.cell.2018.02.012 29502969 PMC6005365

[B28] Jaffer KY, Chang T, Vanle B, Dang J, Steiner AJ, Loera N, Abdelmesseh M, Danovitch I, Ishak WW (2017) Trazodone for insomnia: a systematic review. Innov Clin Neurosci 14:24–34. PMC5842888 29552421

[B29] Jagannath A, Taylor L, Wakaf Z, Vasudevan SR, Foster RG (2017) The genetics of circadian rhythms, sleep and health. Hum Mol Genet 26:R128–R138. 10.1093/hmg/ddx240 28977444 PMC5886477

[B30] Kalmbach DA, Anderson JR, Drake CL (2018) The impact of stress on sleep: pathogenic sleep reactivity as a vulnerability to insomnia and circadian disorders. J Sleep Res 27:e12710. 10.1111/jsr.12710 29797753 PMC7045300

[B31] Keene AC, Duboue ER (2018) The origins and evolution of sleep. J Exp Biol 221:jeb159533. 10.1242/jeb.159533 29895581 PMC6515771

[B32] Killgore WD (2010) Effects of sleep deprivation on cognition. Prog Brain Res 185:105–129. 10.1016/B978-0-444-53702-7.00007-5 21075236

[B33] Kloefkorn H, Aiani LM, Lakhani A, Nagesh S, Moss A, Goolsby W, Rehg JM, Pedersen NP, Hochman S (2020) Noninvasive three-state sleep-wake staging in mice using electric field sensors. J Neurosci Methods 344:108834. 10.1016/j.jneumeth.2020.108834 32619585 PMC7454007

[B34] Kohn M, Litchfield D, Branchey M, Brebbia DR (1974) An automatic hybrid analyzer of sleep stages in the rat. Electroencephalogr Clin Neurophysiol 37:518–520. 10.1016/0013-4694(74)90095-9 4138909

[B35] Kohtoh S, Taguchi Y, Matsumoto N, Wada M, Huang ZL, Urade Y (2008) Algorithm for sleep scoring in experimental animals based on fast Fourier transform power spectrum analysis of the electroencephalogram. Sleep Biol Rhythms 6:163–171. 10.1111/j.1479-8425.2008.00355.x

[B36] Kopp C, Rudolph U, Keist R, Tobler I (2003) Diazepam-induced changes on sleep and the EEG spectrum in mice: role of the alpha3-GABA(A) receptor subtype. Eur J Neurosci 17:2226–2230. 10.1046/j.1460-9568.2003.02651.x 12786990

[B37] Krstajic D, Buturovic LJ, Leahy DE, Thomas S (2014) Cross-validation pitfalls when selecting and assessing regression and classification models. J Cheminform 6:10. 10.1186/1758-2946-6-10 24678909 PMC3994246

[B38] Lee YJ, Lee JY, Cho JH, Choi JH (2022) Interrater reliability of sleep stage scoring: a meta-analysis. J Clin Sleep Med 18:193–202. 10.5664/jcsm.9538 34310277 PMC8807917

[B39] LeGates TA, Altimus CM, Wang H, Lee HK, Yang S, Zhao H, Kirkwood A, Weber ET, Hattar S (2012) Aberrant light directly impairs mood and learning through melanopsin-expressing neurons. Nature 491:594–598. 10.1038/nature11673 23151476 PMC3549331

[B40] Li YD, et al. (2024) Anterior cingulate cortex projections to the dorsal medial striatum underlie insomnia associated with chronic pain. Neuron 112:1328–1341.e4. 10.1016/j.neuron.2024.01.014 38354737

[B41] Lo CC, Chou T, Penzel T, Scammell TE, Strecker RE, Stanley HE, Ivanov P (2004) Common scale-invariant patterns of sleep-wake transitions across mammalian species. Proc Natl Acad Sci U S A 101:17545–17548. 10.1073/pnas.0408242101 15583127 PMC536051

[B42] Lu J, Sherman D, Devor M, Saper CB (2006) A putative flip-flop switch for control of REM sleep. Nature 441:589–594. 10.1038/nature04767 16688184

[B43] Mague SD, et al. (2022) Brain-wide electrical dynamics encode individual appetitive social behavior. Neuron 110:1728–1741.e7. https://doi.10.1016/j.neuron.2022.02.016 35294900 10.1016/j.neuron.2022.02.016 PMC9126093

[B44] Mahoney CE, Cogswell A, Koralnik IJ, Scammell TE (2019) The neurobiological basis of narcolepsy. Nat Rev Neurosci 20:83–93. 10.1038/s41583-018-0097-x 30546103 PMC6492289

[B45] Mao Y, Xu Y, Yuan X (2022) Validity of chronic restraint stress for modeling anhedonic-like behavior in rodents: a systematic review and meta-analysis. J Int Med Res 50:3000605221075816. 10.1177/03000605221075816 35196899 PMC8891861

[B46] Marini G, Imeri L, Mancia M (1988) Changes in sleep–waking cycle induced by lesions of medialis dorsalis thalamic nuclei in the cat. Neurosci Lett 85:223–227. 10.1016/0304-3940(88)90355-2 3374838

[B47] McKillop LE, Fisher SP, Milinski L, Krone LB, Vyazovskiy VV (2021) Diazepam effects on local cortical neural activity during sleep in mice. Biochem Pharmacol 191:114515. 10.1016/j.bcp.2021.114515 33713641 PMC8363939

[B48] Mu P, Huang YH (2019) Cholinergic system in sleep regulation of emotion and motivation. Pharmacol Res 143:113–118. 10.1016/j.phrs.2019.03.013 30894329

[B49] Nestler EJ, Barrot M, DiLeone RJ, Eisch AJ, Gold SJ, Monteggia LM (2002) Neurobiology of depression. Neuron 34:13–25. 10.1016/S0896-6273(02)00653-0 11931738

[B50] Oishi Y, et al. (2017) Slow-wave sleep is controlled by a subset of nucleus accumbens core neurons in mice. Nat Commun 8:734. 10.1038/s41467-017-00781-4 28963505 PMC5622037

[B51] Parks DF, Schneider AM, Xu Y, Brunwasser SJ, Funderburk S, Thurber D, Blanche T, Dyer EL, Haussler D, Hengen KB (2024) A nonoscillatory, millisecond-scale embedding of brain state provides insight into behavior. Nat Neurosci 27:1829–1843. https://doi.10.1038/s41593-024-01715-2 39009836 10.1038/s41593-024-01715-2

[B52] Pronier E, Morici JF, Girardeau G (2023) The role of the hippocampus in the consolidation of emotional memories during sleep. Trends Neurosci 46:912–925. 10.1016/j.tins.2023.08.003 37714808

[B53] Qiu MH, Liu W, Qu WM, Urade Y, Lu J, Huang ZL (2012) The role of nucleus accumbens core/shell in sleep-wake regulation and their involvement in modafinil-induced arousal. PLoS One 7:e45471. 10.1371/journal.pone.0045471 23029032 PMC3446879

[B54] Rayan A, Agarwal A, Samanta A, Severijnen E, van der Meij J, Genzel L (2024) Sleep scoring in rodents: criteria, automatic approaches and outstanding issues. Eur J Neurosci 59:526–553. https://doi.10.1111/ejn.15884 36479908 10.1111/ejn.15884

[B55] Riemann D, Krone LB, Wulff K, Nissen C (2020) Sleep, insomnia, and depression. Neuropsychopharmacology 45:74–89. 10.1038/s41386-019-0411-y 31071719 PMC6879516

[B56] Silber MH, et al. (2007) The visual scoring of sleep in adults. J Clin Sleep Med 3:121–131. 10.5664/jcsm.26814 17557422

[B57] Sriji SN, Akhtar N, Mallick HN (2021) Mediodorsal thalamus lesion increases paradoxical sleep in rats. Sleep Sci 14:33–38. https://doi.10.5935/1984-0063.2090155 34104335 10.5935/1984-0063.20190155 PMC8157787

[B58] Takahashi K, Lin JS, Sakai K (2009) Characterization and mapping of sleep-waking specific neurons in the basal forebrain and preoptic hypothalamus in mice. Neuroscience 161:269–292. 10.1016/j.neuroscience.2009.02.075 19285545

[B59] Talbot A, Dunson D, Dzirasa K, Carlson D (2023) Estimating a brain network predictive of stress and genotype with supervised autoencoders. J R Stat Soc Ser C Appl Stat 72:912–936. 10.1093/jrsssc/qlad035 PMC10474874 37662555

[B60] Tang X, Yang L, Liu X, Sanford LD (2005) Influence of tetrodotoxin inactivation of the central nucleus of the amygdala on sleep and arousal. Sleep 28:923–930. 10.1093/sleep/28.8.923 16218075

[B61] Torquati L, Mielke GI, Brown WJ, Burton NW, Kolbe-Alexander TL (2019) Shift work and poor mental health: a meta-analysis of longitudinal studies. Am J Public Health 109:e13–e20. 10.2105/AJPH.2019.305278 PMC6775929 31536404

[B62] Tsuno N, Besset A, Ritchie K (2005) Sleep and depression. J Clin Psychiatry 66:1254–1269. 10.4088/JCP.v66n1008 16259539

[B63] Ulrich K, Carlson DE, Dzirasa K, Carin L (2015) GP kernels for cross-spectrum analysis. Advances in Neural Information Processing Systems 28 (Nips 2015) 28.

[B64] Viterbi AJ (1967) Error bounds for convolutional codes and an asymptotically optimum decoding algorithm. IEEE Trans Inf Theory 13:260–269. 10.1109/TIT.1967.1054010

[B65] Walder-Christensen K, et al. (2024) Electome network factors: capturing emotional brain networks related to health and disease. Cell Rep Methods 4:100691. 10.1016/j.crmeth.2023.100691 38215761 PMC10832286

[B66] Wang X, et al. (2016) Altered mGluR5-Homer scaffolds and corticostriatal connectivity in a Shank3 complete knockout model of autism. Nat Commun 7:11459. 10.1038/ncomms11459 27161151 PMC4866051

[B67] Welch JM, et al. (2007) Cortico-striatal synaptic defects and OCD-like behaviours in Sapap3-mutant mice. Nature 448:894–900. 10.1038/nature06104 17713528 PMC2442572

[B68] Wilson DJ (2019) The harmonic mean p-value for combining dependent tests. Proc Natl Acad Sci U S A 116:1195–1200. 10.1073/pnas.1814092116 30610179 PMC6347718

[B69] Zhang X, Landsness EC, Chen W, Miao H, Tang M, Brier LM, Culver JP, Lee JM, Anastasio MA (2022) Automated sleep state classification of wide-field calcium imaging data via multiplex visibility graphs and deep learning. J Neurosci Methods 366:109421. 10.1016/j.jneumeth.2021.109421 34822945 PMC9006179

